# Chemistry, properties, and applications of fluorographene

**DOI:** 10.1016/j.apmt.2017.05.004

**Published:** 2017-12

**Authors:** Demetrios D. Chronopoulos, Aristides Bakandritsos, Martin Pykal, Radek Zbořil, Michal Otyepka

**Affiliations:** Regional Centre of Advanced Technologies and Materials, Department of Physical Chemistry, Palacký University Olomouc, tř. 17. listopadu 12, 771 46 Olomouc, Czech Republic

**Keywords:** Fluorographene, Chemistry, Graphene derivatives, Covalent functionalization

## Abstract

Fluorographene, formally a two-dimensional stoichiometric graphene derivative, attracted remarkable attention of the scientific community due to its extraordinary physical and chemical properties. We overview the strategies for the preparation of fluorinated graphene derivatives, based on top-down and bottom-up approaches. The physical and chemical properties of fluorographene, which is considered as one of the thinnest insulators with a wide electronic band gap, are presented. Special attention is paid to the rapidly developing chemistry of fluorographene, which was advanced in the last few years. The unusually high reactivity of fluorographene, which can be chemically considered perfluorinated hydrocarbon, enables facile and scalable access to a wide portfolio of graphene derivatives, such as graphene acid, cyanographene and allyl-graphene. Finally, we summarize the so far reported applications of fluorographene and fluorinated graphenes, spanning from sensing and bioimaging to separation, electronics and energy technologies.

## Highlights

• Synthesis and properties of fluorographene and fluorinated graphenes are introduced.
• Both experimental and theoretical approaches are amalgamated in order to provide a coherent insight.
• Toxicity and biocompatibility of fluorographene is discussed.
• Recent advanced in chemistry of fluorographene are thoroughly overviewed.
• Applications of fluorographene and fluorinated graphenes are summarised.

## Graphical abstract

**Figure:**
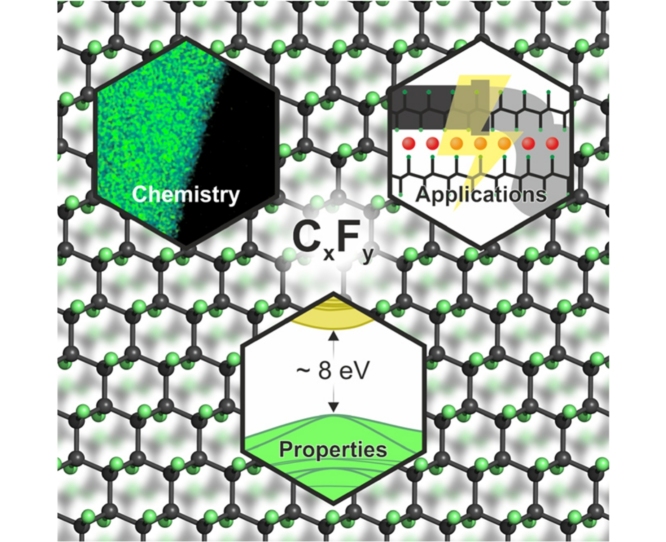

## 1 Introduction

Graphene, which is an atomically thin layer of *sp*^2^ carbons arranged into a honeycomb lattice [1], caused revolution in materials science [2], [3], [4], [5], [6], [7]. Graphene is formally a two-dimensional (2D) material having extremely high surface-to-mass ratio. It displays numerous physical features, which can be exploited in a large number of applications [8], [9], [10], [11], [12], [13], [14], [15], [16], [17], [18], [19], [20], [21], [22]. On the other hand, graphene is a hydrophobic material not compatible with water polar medium, which limits its application potential, such as in sensing and bioapplications. Graphene properties can be modified by its covalent and noncovalent functionalization. The covalent functionalization seems to be vivid strategy to modulate graphene’s physicochemical properties [7]. This calls for establishing facile and robust chemistry of graphene in order to broaden its application potential. Unfortunately, graphene itself is rather chemically inert material and harsh reaction conditions have to be applied to directly modify graphene. The so far established chemistries lead to graphene derivatives with a low degree of functionalization (typically 1–3%) [23], [24], [25], [26], [27] or to derivatives with complex chemical nature, like in the case of graphene oxide [28].

Fluorographene (FG) was predicted as a stable graphene derivative by Sofo and coworkers in 2007 [29] and prepared by fluorination of graphene [30], [31] and mechanical [31] or chemical [32] exfoliation of graphite fluoride three years later. FG was considered the thinnest insulator and counterpart of polytetrafluoroethylene (Teflon ^®^) [31], because it is perfluorinated hydrocarbon. Since its discovery, physical and chemical properties of fluorographene have been thoroughly analyzed. Numerous studies show that FG is not chemically inert and undergoes various chemical reactions under ambient conditions. The chemistry of fluorographene is now a budding discipline because it can lead to various graphene derivatives and represent efficient strategy for synthesis of tailored graphene derivatives with a high degree of functionalization [33], [34].

## 2 Synthesis of fluorinated graphene and fluorographene

Preparation of fluorinated graphene or fluorographene is mainly based on two strategies: (a) liquid-phase or mechanical exfoliation of bulk graphitic materials containing fluorine atoms, such as the commercial graphite fluoride (GrF) and (b) fluorination of graphene layers with the aid of fluorinating agents.

### 2.1 Exfoliation

**Fig. 1:**
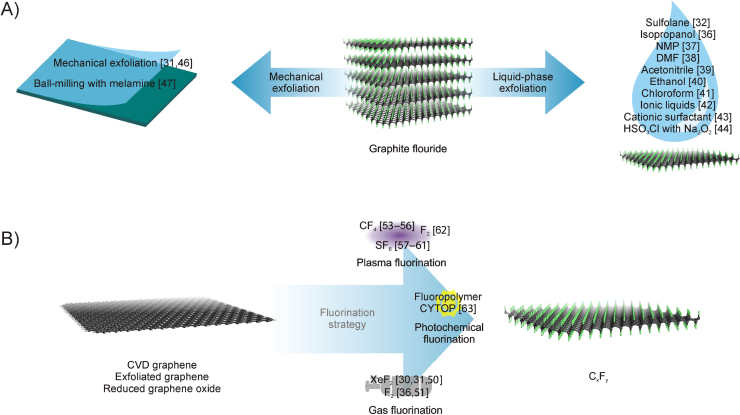
Illustration of A) exfoliation strategies for the preparation of fluorinated graphenes and, B) fluorination approaches for the preparation of fluorinated graphenes.

Exfoliation of multilayer materials is a well-established approach, which is successfully applied, e.g., for production of graphene nanosheets. In the liquid-phase exfoliation process, a solvent is used as an intercalating agent, weakening the van der Walls interactions [35] between the neighboring fluorographitic layers and resulting in the exfoliation of single or few-layer fluorographene from the pristine material (Fig. 1A). Regarding FG, Zbořil et al. applied this method for the first time [32]. Bulk GrF was suspended in sulfolane and the mixture was sonicated at 50 °C for 1 h, providing single layers of stoichiometric C_1.0_F_1.0_ FG. At the same time, Cheng et al. [36] employed isopropanol, receiving multilayers of graphene fluoride by sonochemical exfoliation of fluorinated pyrolytic graphite. Afterwards, many works were reported, concerning the preparation of FG, based on the above-mentioned strategy. A significant number of organic solvents, such as *N*-methyl-2-pyrrolidone (NMP) [37], *N,N*-dimethylformamide (DMF) [38], acetonitrile [39], ethanol [40], and chloroform [41], has been successfully applied in that context so far. Besides organic solvents, intercalation has been achieved using molecules such as ionic liquids [42], cationic surfactant (cetyl-trimethylammonium bromide, CTAB) with dopamine [43], and sodium peroxide (Na_2_O_2_) with chlorosulfonic acid (HSO_3_Cl) [44]. At that point, it should be noted that some solvents, such DMF and NMP, can cause partial defluorination of FG during the ultrasonication [45].

Mechanical exfoliation (Fig. 1A) represents another strategy for preparation of FG [31], however, such approach is hardly scalable and is suitable namely for fine physical experiments. It was demonstrated that fully and partially fluorinated graphenes can be mechanically exfoliated from highly oriented pyrolytic graphene (HOPG), which was fluorinated by F_2_ and XeF_2_ to 24% and 100% degree of fluorination [46]. Very recently, ball-milling conditions with the aid of melamine were applied for the exfoliation of FG layers from GrF [47].

Finally, another reported method for delamination and expansion of GrF is thermal exfoliation. A partially fluorinated graphite was thermally exfoliated by inserting the solid sample in a preheated furnace at 800 °C, and further used for film formation and electrochemical testing [48]. Thermal exfoliation of GrF was studied in more details by Jankovský et al. [49]. Commercial GrF treated at 400 °C contained 49 at.% of F, while treatment at 600, 800 and 1000 °C, resulted to products with ∼9, 7, and 3 at.% of F, respectively. The exfoliation was effective at temperatures above *ca.* 600 °C, as SEM analysis revealed.

### 2.2 Fluorination

Fluorination strategies are based on the model by which graphene [e.g., exfoliated graphene, graphene grown by chemical vapor deposition (CVD), reduced graphene oxide (RGO)] is placed into a reactor with fluorination agents, which are used as precursors for the generation of active intermediates with the aid of either thermal, pressure, or irradiation initiation (Fig. 1B). These intermediates attack the *sp*^2^-hybridized carbons of graphene lattice, forming C-F *sp*^3^ bonds and producing fluorinated graphenes. For the direct gas-fluorination of graphene, one of the most common fluorinating agents is xenon difluoride (XeF_2_) gas and it was utilized for the fluorination of graphene at various temperatures under an inert atmosphere [30], [31], [50]. According to room-temperature synthesis of FG, graphene grown by CVD on silicon-on-insulator substrate was fluorinated by XeF_2_, providing a stoichiometric material C_1.0_F_1.0_ fully fluorinated on both sides, due to the effective etching on the Si substrate by reactive gas [30]. Fluorine (F_2_) gas, another important fluorination agent, was applied to fluorinate graphene and graphene derivatives at various temperatures and pressures [36]. Moreover, RGO was converted to FG via treatment with F_2_ [51]. It should be noted that the support of graphene determines if the graphene is one or both sides functionalized and affects stability of the fluorinated graphene [52].

Plasma fluorination is an alternative way for fluorination of graphene, targeting to the synthesis of controllable fluorinated graphene materials. According to this technique, generated fluorine radicals adsorb onto carbon lattice and create different C-F bonds. The fluorine-based compounds CF_4_ [53], [54], [55], [56], SF_6_ [57], [58], [59], [60], [61], and F_2_ [62] represent the most common plasma sources. Photochemical fluorination is an innovative and ecofriendly method for the production of fluorinated graphene. Molecules containing fluorine are used as precursors for the generation of fluorine radicals with the aid of irradiation. According to this approach, fluoropolymer CYTOP coated the surface of a single-layer graphene film on a SiO_2_/Si substrate and then, it decomposed under laser irradiation, generating active fluorine radicals, which reacted with carbon lattice and formed C-F bonds, providing fluorinated graphene C_4_F [63]. Low degree of fluorination (∼1.4 at.%) was also attained through solution processing of graphite with BrF_3_ in liquid Br_2_ [48], [64]. In the following, the fluorinated graphite derivative was exfoliated via thermal exfoliation [48] or by ultrasonication and centrifugal fractionation [64].

All the methods discussed above employ graphene or bulk GrF for the preparation of fluorinated graphene or fluorographene. However, in the last years, there has been an increasing number of publications regarding the fluorination of GO, synthesizing fluorinated graphene oxide (FGO). Direct gas-fluorination has been successfully applied to the fluorination of GO, using as precursors F_2_ [65], [66] or other fluorine-based compounds such a SF_6_, SF_4_, and MoF_6_ [67], in order to avoid the high toxicity of F_2_ and to improve the controllability of the C-F bonding character. At that point, it is worth mentioning that the approach above has been also used for the preparation of fluorographane through treatment of graphane with F_2_ [68]. Moreover, as in the case of graphene, GO has been fluorinated via plasma method [69] or photochemical fluorination [70]. Fluorination of GO has been achieved by hydrothermal or solvothermal fluorination, since GO has many oxygen-containing groups, which can be transformed to C-F bonds at high temperature, using precursors such as hydrofluoric acid (HF) [71], [72], [73], BF_3_-etherate [74], diethylaminosulfur trifluoride (DAST) [75], [76], and hexafluorophosphoric acid (HPF_6_) [77]. Furthermore, preparation of FGO was accomplished via an electrochemical method [78].

## 3 Properties

**Fig. 2:**
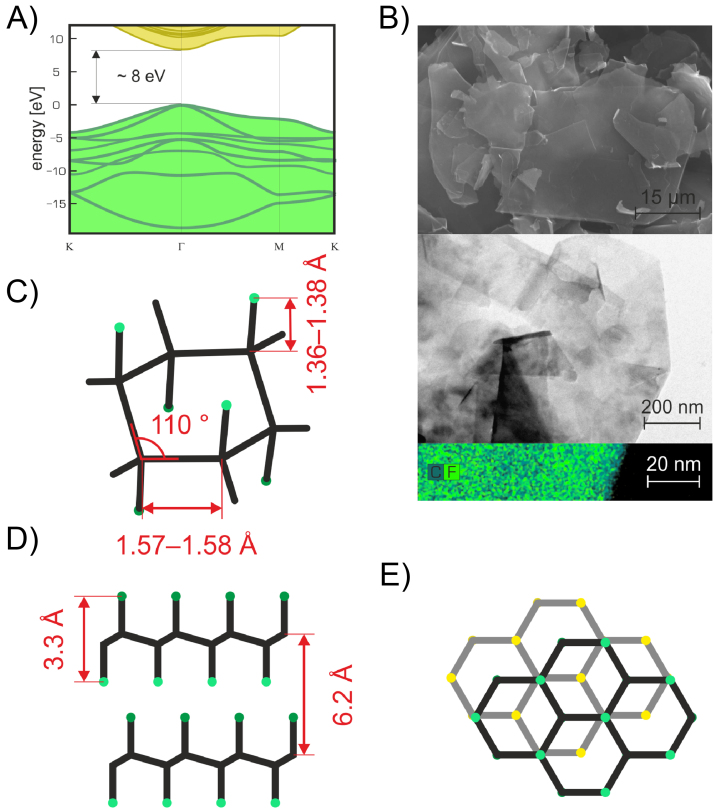
A) The electronic band structure of FG in the vicinity of the band gap, which is predicted to amount up to 8 eV. B) SEM (upper part), TEM (middle part), and carbon and fluorine EDS maps (bottom part) images of FG. C) Geometrical features of the hexagonal unit of FG. D) The distance between the F–F planes in FG (3.3 Å) and the interlayer distance between two FG layers (6.2 Å), E) AB stacking motif of FG in graphite fluoride.

The incorporation of fluorine atoms onto carbon lattice and the C/F (C *sp*^2^/*sp*^3^) ratio significantly affect the electronic and optical properties of fluorinated graphenes. Already a small amount of fluorine atoms in the structure give rise to a band gap opening [30]. The absorption spectrum of FG demonstrated its transparency in the range of visible light [31]. Additionally, the spectrum showed that FG started absorbing light only in the blue region (energy >3.0 eV), indicating that FG is a wide-gap semiconductor or an insulator with a wide band gap ≥3.0 eV [31]. Estimation of electronic band can also be achieved by using theoretical calculations. Whereas various methods are consistent in prediction of the shape of the electronic band structure indicating FG as a direct band gap material with valence band maximum and conduction band minimum situated in the Γ point in the first Brillouin zone, the absolute values of the band gap vary depending on the used method and level of theory. It should be noted that the values are generally calculated for the most stable chair conformation of fluorographene with C_1_F_1_ ratio, whereby other geometrical configurations differ only slightly from values calculated for the chair conformation [79]. The lowest band gap values (3.0–4.2 eV) were obtained using standard LDA (local density approximation) and GGA (generalized gradient approximation) approximations in density functional theory (DFT) [46], [80], [81], [82], [83]. However, any agreement of LDA and GGA estimates with the optical measurements is purely coincidental. Generally, these estimates cannot be directly comparable with experimental data of optical transitions of FG, because they do not consider excitonic effects (electron–hole interaction). It was shown that both LDA and GGA approaches systematically underestimate the electronic band gap [84]. On the other hand, it has been shown that hybrid functionals provide reliable band gap estimates for some solids and carbon materials [85]. In the case of fluorographene screened hybrid HSE06 functional [86] a band gap of 5.1 eV was predicted [83]. The high-level many-body calculations using the GW approximation (GWA) estimated a broader electronic band gap (Fig. 2A) up to 7.0–8.3 eV [79], [87], [88].

In order to study photoluminescence (PL) of FG, material dispersion in acetone was excited by 290 nm at room temperature [50]. The spectrum exhibited two emission peaks at around 3.8 eV and 3.65 eV, indicating the formation of a wide bandgap semiconductor [50]. According to the attribution of the peaks, the first one was associated with the band-to-band recombination of free electrons and holes, while the same energy was measured for the band gap of FG by near edge X-ray absorption fine structure spectroscopy (NEXAFS) [50]. The latter peak (156 meV below the bandgap emission) was ascribed to phonon-assisted radiative recombination across the band gap, for which the C—F vibration mode was excited when the electron–hole pair recombined. In the same context, FG containing a lower amount of fluorine was also measured, resulting in the appearance of two accompanying peaks at 2.88 eV and 2.73 eV [50]. For a theoretical description of absorption and emission spectra, it is necessary to consider also the electron–hole correlation effects. This can be achieved by solving Bethe-Salpeter equation (BSE) on top of GWA [88], [89]. Calculations at the BSE + G_0_W_0_@PBE level for FG showed an excitonic peak at 5.14 eV [84]. This calculation identified rather unusually high exciton binding energy of 1.85 eV. It is worth noting that the position of the first excitonic peak was relatively insensitive to number of layers and amounted to 5.20 eV for GrF, which was in a good agreement with optical measurements on GrF [90]. However, the experiments indicate rather lower value of FG band gap ∼3 eV (see above). Yuan and coworkers ruled out that this discrepancy could stem from structural defects, which only slightly reduce the band gaps [91]. Hence, the discrepancy between the experimental and calculated band gap of FG remain unanswered and certainly deserves a further analysis (especially taking into account a role of trions) [92].

Another significant proof for the optical transparency of FG is the absence of Raman signals. That happens because the energies of the lasers, which are used for Raman spectroscopy, are lower than the band gap [30], [31]. In order to overcome this problem, Wang and coworkers [90] used a UV laser (energy 5.08 eV), detecting two Raman active modes at 1270 cm^−1^ and 1345 cm^−1^. According to Fourier transform infrared (FTIR) spectroscopy, FG exhibits a strong band at 1260 cm^−1^, which is attributed to the covalent C—F bond stretching [50], [69]. This FTIR fingerprint of C—F bond can be effectively used for direct monitoring of the progress of reactions involving FG [33], [34]. Additionally, FG exfoliated by NMP displays two bands at 1212 cm^−1^ and 1084 cm^−1^, corresponding to the stretching vibration of the C—F covalent bonds and the semi-ionic stretching vibration of C—F bonds, respectively [37]. Further studies of FTIR spectra depended on fluorination time showed that the weak band at 1112 cm^−1^, ascribable to the semi-ionic C-F bond, progressively transforms to covalent C—F bond, which is exhibited as a strong band at 1211 cm^−1^ [93]. All the experimental infrared active modes for the C—F stretching bond are in good agreement with those theoretically calculated [94]. Moreover, Ueta et al. [95] showed in molecular dynamics (MD) simulations that the IR spectrum polarized along the graphene surface direction could resolve more bands, according to the arrangement of fluorine atoms on the lattice. One fluorine atom on the inner graphene surface displayed a signal at 800 cm^−1^, due to the local wagging motion of the C-F bond. In the case of fluorine dimer, IR signal appeared at 1150 cm^−1^, originated by the local stretching motion of the bond formed by the C—C atoms connected to the fluorines.

X-ray photoelectron spectroscopy (XPS) is a powerful technique providing information about elemental composition and binding conditions of elements in FG. According to XPS data, C—F type was the major bond (86%) in this material, while smaller fractions of C-F_2_ (12%) and C-F_3_ (2%) species were also present, owing to defects at free edges [30]. The carbon peak was observed at ∼287.5 eV, assigned to a C-F binding state. Later, the presence of various C-F functionalities was confirmed and attributed to C—CF (∼286.9 eV), CF—CF_2_ (∼289.9 eV), C—F_2_ (∼292.1 eV), and C—F_3_ (∼293.7 eV) [71]. Concerning F 1s spectra, the C-F bonding type was gradually shifted from 685.5 eV (semi-ionic) to 687.5 eV (covalent), increasing the fluorination time [93]. Studying the chemical bonding of FG, it was demonstrated that the character of the C—F bond depends on local concentration and arrangement of the fluorine species, reacting on the bonding states of fluorine on graphene and forming semi-ionic, covalent, and intermediates bonds [96]. Additionally, theoretical calculations showed that the C-F bond strength varies with the fluorine content in the structure of FG. In systems having low content of fluorines the dissociation energy of C—F bond is low reaching to 49.6 kcal mol^−1^, while in the fully fluorinated structure it amounts to 112.3 kcal mol^−1^ [97], which is comparable with C—F bond energies of organic compounds [98]. ^19^F magic angle spinning (MAS) NMR studies on fluorinated graphite showed that the strength of the C—F bond, or its ionic/covalent character, could be also probed with NMR, according to which lower chemical shifts point to higher covalent character [99].

Scanning and transmission electron microscopy (SEM and TEM) provide significant information about the layered morphology of FG (see Fig. 2B), while TEM proved the transparent nature of FG (i.e., single or few-layer structure) with lateral dimensions in a range between 200 nm and 2 μm [31], [32]. Additionally, the existence of a hexagonal crystalline structure [31], [32], [36] and stoichiometry [32] equivalent to that of bulk GrF were confirmed by the aid of selected area electron diffraction (SAED) analysis. It was observed that the retention of hexagonal crystalline order for FG is similar to that of graphene with 1% expansion of the unit cell [31], [36], while the experimental lattice constant (α) of C_1_F_1_ was found to be 2.48 Å, slightly larger than for graphene (2.46 Å). The value of the lattice parameter is in good agreement with the theoretical calculations (α = 2.48 Å), and it corresponds to the chair conformation with AA stacking sequence, the most stable structure [35]. The increase of FG’s lattice constant was expected because fluorination creates *sp*^3^ C—C bonds, which are larger than the *sp*^2^-type bonding of graphene. The C—C and C—F bond lengths and the repeating unit of FG are shown in Fig. 2C. However, according to a recent molecular dynamics study, the FG structure in contrast with graphene remains substantially undistorted even at higher temperatures [100]. For the estimation of the thickness of a single FG layer, atomic force microscopy (AFM) was employed, determining that the thickness is in a range of 0.67–0.87 nm [32]. The experimental values were confirmed by theoretical calculations, which provided the thickness of monolayer and two-layer FG 0.62 nm and 1.24 nm, respectively [32] (see Fig. 2D). The individual layers in GrF are bound primarily by van der Waals interactions, arranged in a AB stacking sequence (see Fig. 2E), whereby the energy needed for exfoliation of FG is about 190 mJ m^−2^ [35]. Concerning the surface properties of FG, recent studies by inverse gas chromatography revealed that GrF has a lower surface energy (of ∼80 mJ m^−2^) than graphite [35]. Additionally, the adsorption enthalpies of organic molecules are similar or even slightly lower to FG than to graphene [101], [102].

In order to utilize FG for bioapplications, it was necessary to examine its biocompatibility and potential toxicity in cells. For that reason, extensive studies were carried out [93], [103], [104], [105], [106]. It was shown that fluorinated graphene enhanced adhesion, proliferation and polarization of mesenchymal stem cells [90], which predetermines FG as a suitable scaffold for tissue-engineering. Recent studies showed that FG with larger size and higher amounts of fluorine atoms imparted higher toxicological effects on A549 cells [105], [106].

## 4 Chemistry

Regarding the chemical behavior of FG, it was considered as an inert material, presenting similar properties to GrF and polytetrafluoroethylene (Teflon ^®^), such as chemical stability in many solvents and high hydrophobicity [31]. Additionally, FG is quite thermally stable, starting to decompose at temperature range 300–400 °C. At higher temperatures (400–600 °C), low-molecular-weight volatile C_x_F_y_ products evolve from the material [30]. Despite the fact that FG is considered as a chemically inert material containing C-F bonds, which belong to the strongest single bonds in organic compounds [98], [107], scientists showed from the first publications that FG can undergo reductive defluorination, resulting in the formation of graphene [38]. This unusual cleavage of fluorine atoms may be explained through the admission that C—F bond of FG has a semi-ionic character [88]. Further theoretical studies showed that FG is susceptible to nucleophilic substitution [97], opening an avenue for the development methods for its covalent modification and the preparation of graphene derivatives.

As already mentioned, defluorination of FG is an important approach for its transformation to graphene. For that aim, a significant number of methods have been developed. They are mainly classified into two groups: (a) chemical and (b) thermal reduction. Robinson et al. [30] studied the two aforementioned strategies and found that the chemical defluorination of FG was more effective than the thermal reduction. According to this work, defluorination was carried out by exposure of FG to hydrazine vapors, restoring the conductivity and ambipolar nature of graphene 4CF_n_ + nN_2_H_4_ → 4C + 4nHF + 2N_2_. At the same period, our group [32] reported an alternative way for the defluorination of FG, using KI in DMF. In this reaction, FG was transformed into unstable graphene iodine, which quickly decomposed to graphene and iodine at 150 °C, following the equation: CF + KI → KF + CI → C_(graphene)_ + KF + 1/2 I_2_. Additionally, graphene was produced by reduction of FG, utilizing either triethylsilane or zinc nanoparticles [38]. Moreover, the selective reduction of semi-ionic fluorinated graphene (s-FG) was achieved after treatment with acetone via the process 2C_2_F_(semi-ionic)_ + CH_3_C(O)CH_3(l)_ → HF + 2C_(s)_ + C_2_F_(covalent)_ + CH_3_C(O)CH_3(l)_ [108]. The reduction of s-FG was not feasible when methanol and water were used for the same aim. Beyond the above-mentioned approaches for the defluorination of FG, Ren et al. reported that ultraviolet irradiation and aromatic solvents like toluene act synergistically for the partial defluorination of FG, controlled by the irradiation time [109].

**Fig. 3:**
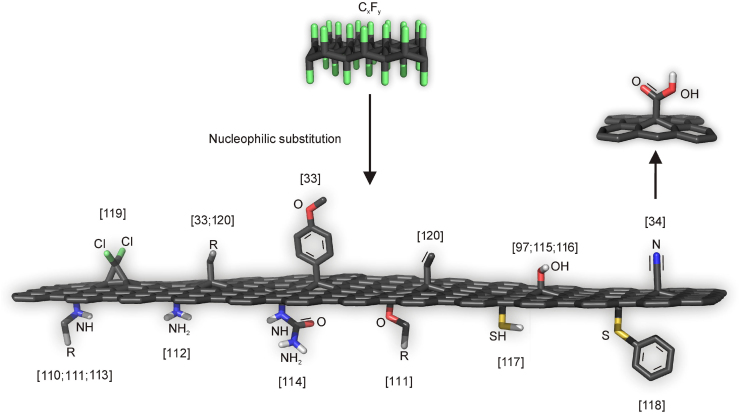
Reactions of fluorographene lead to a wide portfolio of graphene derivatives.

Regarding the nucleophilic substitution of FG and the preparation of covalent functionalized graphene derivatives, N-, O-, S- and C-nucleophiles have been applied for that purpose (see Fig. 3). Ethylenediamine was the first nucleophilic compound, which was employed for the covalent functionalization of FG [110]. Since then, many groups investigated the reaction between amine-bearing compounds and FG [111], [112], [113], [114]. In particular, Whitener et al. [111] extensively studied the electrophilic character of FG through reactions between FG with various of amine-, alcohol- and sulfur-bearing compounds, which have high nucleophilicity. The results showed that amines and alcohols reacted successfully with FG, providing covalent functionalized graphene derivatives. Very recently, two new graphene derivatives dispersible in water were prepared based on the context above, amino-fluorographite (AFGr) [112] and urea-modified FG (UFG) [114], after treatment of FG with sodium amide and urea, respectively. As already mentioned above, alcohols can be used as nucleophiles for the modification of FG [111]. Moreover, experiments showed the process of nucleophilic exchange of fluorine atoms at FG by hydroxide ions [97], [115], [116], preparing hydroxylated graphene. Sulfur nucleophiles (e.g., thiols, sulfonic acids) act more effectively as reducing agents than as nucleophiles, displacing fluorine atoms from FG without substitution and providing reduced FG [111]. In order to explain this fact, authors suggested a mechanism based on the ability of thiols to self-react forming disulfides. It was, however, shown that FG treated by NaSH can produce thiofluorographene [G(SH)F] [117], a new hydrophilic graphene derivative. The sulfhydryl groups were stabilized on the surface of the almost defluorinated graphene by the presence of fluorine atoms. Thiofluorographene was tested as biosensor, demonstrating remarkable results for the impedimetric detection of DNA hybridization. The successful covalent modification of FG by thiophenol was also proved in the gas phase [118].

On the other hand, the modification through the formation of C—C bonds between carbons of FG and carbons of the organic units had not received the proper attention and only the last years some remarkable works were reported. In the first work, chlorocarbene, generated in situ by chloroform after treatment with NaOH, attacked electrophilic carbons of FG, detaching fluorine atoms from FG and forming chloro-terminated cyclopropanic rings with the newly formed *sp*^2^ carbons of the graphene lattice, according to [1 + 2] cycloaddition reaction [119]. Knowing that organometallic reagents contain carbanions, very recently, two publications presented the covalent modification of FG by Grignard reagents [33], [120]. Our group developed an efficient method for the high-degree, homogeneous, and double-sided alkylation and arylation of exfoliated FG, under mild reaction conditions [33]. Additionally, theoretical calculations demonstrated that the value of nucleophilicity plays an important role to the success of the nucleophilic substitution onto FG. According to Mazanek’s work [120], fluorinated thermally reduced graphene reacted with ethyl, vinyl, ethynyl, and propargyl groups, providing graphene derivatives, useful for further chemical modifications. It is well-known that pseudohalides can also react as nucleophiles. Based on that, our group presented that cyano group (-CN) can substitute fluorine atom of FG, synthesizing the cyanographene [34]. The consequential acidic hydrolysis of it resulted in the formation of graphene acid, the first 2D carboxylic acid. Moreover, the carboxyl groups were used for the coupling with a variety of amines via carbodiimide chemistry.

## 5 Applications

GrF and organosiloxane derivatized GrF were studied as solid lubricants and lubricant additives since ’60 s and ’70 s [121], [122], [123], in place of other 2D materials such as graphite and MoS_2_. At that time, there were no reports on the use of single sheets of FG. Nevertheless, the layered nature, low surface energy, and facile exfoliation of GrF plays a significant role in lubrication [124]. GrF, with theoretical specific capacity for Li of 865 mAh g^−1^ [125], which is substantially more than that for pure graphene (372 mAh g^−1^ [126]), was also utilized as cathode material for primary and secondary Li-ion batteries, improving the discharge and shelf-life characteristics of the battery cells [127], [128]. An overview of many other properties and application of GrF, e.g., in batteries, is given in a book [129].

In the case of batteries as well, intercalation and even reactions (i.e., defluorination and transformation of GrF to conductive carbon [127]) take place in between the single layers. Therefore, eventually, the mechanisms in these processes exploit the properties of FG and not of the bulk GrF material only. Application of FG in batteries still provokes research interest [130], [131], [132], [133], [134], [135]. Recently, the application of FG was extended as a very effective electrode separator in Li-sulfur batteries in order to prevent the migration of polysulfides to the Li anode [136], and as an effective cathode in Mg batteries [137]. The interesting electrochemical properties of FG initiated its study as electrode material for supercapacitors [48]. It is suggested that extensive fluorination leads to C—F groups that are electrochemically inactive [138], while the appropriate control of F content can boost the electrochemical performance of FG electrodes [139] or hybrid FG electrodes [138].

FG has been also applied for the electrochemical sensing of ascorbic acid and uric acid and favorably compared to pristine graphene. It was found that by increasing fluorination up to a value of CF_0.75_, the performance was improved with respect to both the linearity of the electrode’s response and the resolution of the oxidation peaks of the two molecules in their mixtures [140]. The applicability of FG as a biosensor was further established by electrochemical detection of NADH and dopamine [141]. Taking advantage of the rich FG’s chemistry, its covalent functionalization with thiol groups resulted in effective DNA impedimetric sensors, based on the interactions developed between the DNA strands and the thiol groups [117]. Very interestingly, such thiol graphene derivatives were thermodynamically stable only in the presence of fluorine adatoms on the graphene skeleton, as density functional theory calculations suggested. Sensing in the gas phase appeared to be another fruitful application area for FG derivatives. Partially fluorinated FG (thus endowed with conductivity) was reported to present interactions with NH_3_ and NO_2_ gas molecules of appropriate strength [64], which allowed both the sorption of the molecules on the film surface, and the regeneration of the sensor by simple Ar purging, without heating or application of vacuum. On the contrary, introduction of oxygen containing groups on the graphene film, resulted to higher binding energies, prohibiting regeneration of the surface.

The applications of FG were extended to solar cell technologies by Das et al. [142] in 2011. It was reported that fluorination of graphene resulted in an increase of the catalytic sites for iodine reduction (an important process taking place in dye sensitized solar cells, DSSC), thus the FG modified electrode performed better as a counter electrode in DSSC than its graphene counterpart. Enhanced power conversion efficiency was later reported by edge functionalization of graphene sheets [131], although direct comparisons are difficult to be made due to differences in the fluoride content, in the flake dimensions, and in the type of dyes used in each work.

**Fig. 4:**
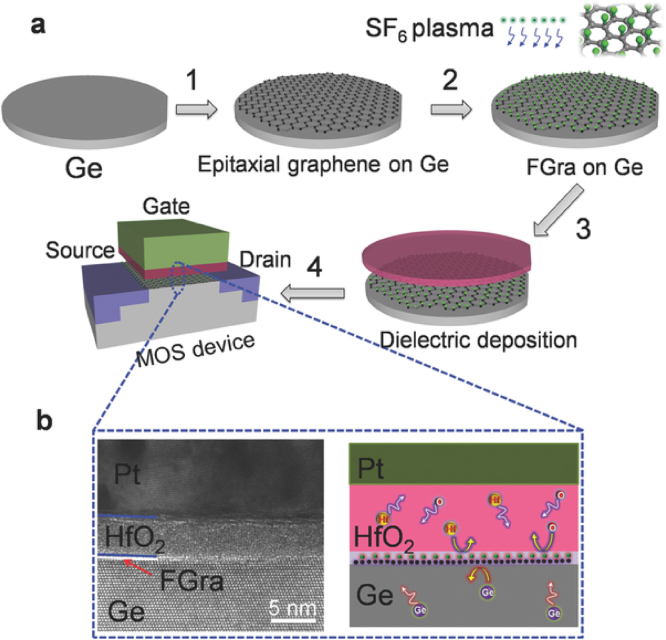
Illustration showing the Ge-based metal-oxide-semiconductor (MOS) device simultaneously utilizing the fluorinated graphene as diffusion barrier and dielectric layer. (Reprinted with permission from Zheng et al. [145] Copyright 2015: Wiley-VCH Verlag GmbH & Co.).

FG, considered as one of the thinnest 2D insulators, is also explored in electronic applications as gate dielectric material and modified layer in organic field effect transistors (FETs) [41], [143]. FG was also successfully used as a passivation layer in self-aligned graphene transistors improving their performance [144]. The possibility to tune graphene’s band gap with fluorination has been exploited in the fabrication of single atomic layer transistors [55]. Selective fluorination after appropriate masking of CVD grown graphene resulted into a single graphene sheet with areas having different band gaps (and thus conductivities), with each area on the sheet having functionality depending on its band gap. FG has been also recently used in Ge-based nanoelectronics due to its insulating and, at the same time, diffusion barrier properties, solving the problem of Ge oxidation, after coating the Ge substrate with epitaxial graphene and subsequent fluorination [145] (Fig. 4).

FG has also potential in electrocatalytic applications. Theoretical studies suggest that partially fluorinated graphene can act effectively as an efficient metal-free catalyst for oxygen reduction reaction (ORR) [146], and two reports experimentally show that F-doping of graphene (along with other dopants) promotes the catalytic activity for oxygen and hydrogen evolution reaction [147], [148]. Moreover, a very recent work presented that the amount of F doped on the RGO has an effect on the ORR electrocatalytic performance [149], while thermally exfoliated GrF at 1000 °C has been identified as excellent ORR metal free catalyst [49]. It is suggested that covalent functionalization of graphene with F introduces spin and charge density, which in turn induces high catalytic activities [148], [150].

Theoretically it has been indeed predicted that spin density can emerge in FG and be modulated by changing the degree of F coverage [151]. Nair et al. [152], Makarova et al. [153], and Feng et al. [154] showed experimentally that fluorine adatoms result to the appearance of spin centers, although different trends were reported regarding the number of spins and F content, as well as regarding the appearance and type of magnetic ordering. Owing to these properties, fluorine and hydrogen functionalized graphene and graphene nitride derivatives are explored as tunnel barriers for spin and charge transport, being thus candidates in future spintronic devices [155], [156]. The chemistry of FG has been recently exploited towards development of room temperature magnetically ordered 2D systems, with very high saturation magnetization of ca. 1 emu g^−1^. This was achieved through preparation of hydroxyl-substituted fluorographene [116], where the simultaneous reductive defluorination and nucleophilic substitution with —OH groups lead to a system containing aromatic islands forming diradicals that communicate through —OH enabled superexchange interactions. These developments in *sp*^2^-based magnetism in 2D systems and particularly room temperature sustainable magnetic ordering, pave the way for the future organic spintronic applications.

**Fig. 5:**
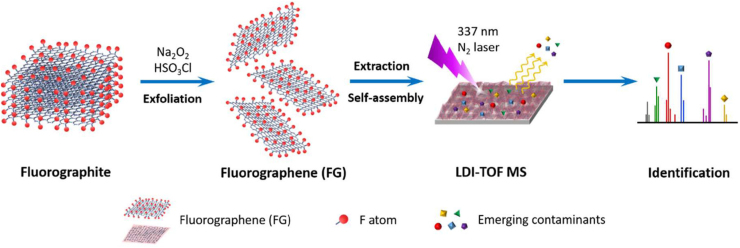
FG acting as a probe in laser desorption/ionization time-of-flight mass spectrometry. (Reprinted with permission from Huang et al. [157] Copyright 2017: American Chemical Society.).

**Fig. 6:**
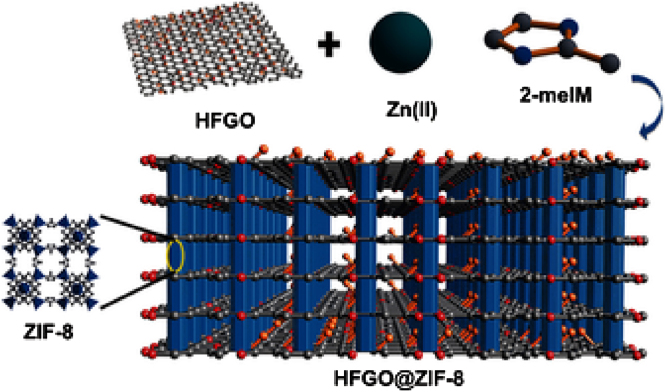
Schematics illustrating the structure of HFGO@ZIF-8 composite, fluorinated graphene oxide pillared by the zeolite. (Reprinted with permission from Jayaramulu et al. [159] Copyright 2016: Wiley-VCH Verlag GmbH & Co.).

Owing to its hydrophobic surface properties, FG found application for anti-corrosion and self-cleaning coatings [158]. Due to the same properties it was also exploited in analytical and environmental related processes. It was shown that FG can be used as a powerful matrix/probe in matrix-assisted or surface-enhanced laser desorption/ionization mass spectrometry for the detection of trace amounts of emerging chemical compounds [157] (Fig. 5). There is also potential in the oil-water separation [159] (Fig. 6). In particular a hybrid system was developed composed of nanocrystalline zeolite imidazole framework ZIF-8 and highly fluorinated graphene oxide, displaying an exceptionally high water contact angle and revealed very high sorption selectivity, fast kinetics, and good sorption properties for nonpolar organic solvents and oils from water. On the other hand, separation technologies for gases have been only theoretically proposed. For example, effective membranes for selective diffusion of CO_2_ and its separation from N_2_ have been predicted by molecular dynamic simulations upon fluorination of a pore rim in graphene [160]. In another theoretical study [161], porous fluorinated graphene is suggested to modulate the heat of adsorption of molecules, enhancing the binding of dipolar ones (H_2_O, SO_2_, H_2_S, and CO_2_) over N_2_, O_2_, and CH_4_. Therefore, applications are envisioned on separation of CO_2_ and SO_2_ from flue gases, purification of natural gas, and removal of H_2_O from air. A significant advantage is predicted for porous FG, related to the fact that gas molecule separation is not dependent on size-exclusion mechanism, but on interaction strengths. Therefore, there is no need for contiguous and unbroken films, but only large accessible surface area is required. In the same area of environmental and separation applications, fluorination of edge carbon atoms of a pore in a graphene sheet has been found by MD simulations very advantageous for extremely high water flux rates and very efficient salt rejection [162]. In contrast, passivation with N atoms instead of F leads to high rejection of both water molecules and ions, owing to the stronger interactions developed. Thus, another application opportunity for FG resides in desalination technology.

FG has also found applications in the field of biomedicine. In 2012, Wang et al. [93] studied the differentiation of the mesenchymal stem cells on fluorinated graphene surfaces, identifying that highly polarized C-F bonds can promote differentiation towards neuronal lineages, thus suggesting FG as a scaffold for controlled tissue engineering. One year later, fluorinated GO was reported as a metal-free contrast agent in magnetic resonance imaging [103], taking advantage of the paramagnetic properties of fluorinated GO [163] and FGO dots [164]. FGO dots show also great potential in cellular imaging because they display enhanced photostability at different pH values, as well as against singlet oxygen, which may evolve during excitation [165].

Finally, in 2012, two independent reports appeared studying the non-linear optical properties of FG [166], [167] and FGO with the open-aperture Z-scan technique. Important third order non-linear optical response was recorded in aqueous colloids of FG, prepared by exfoliation of GrF with the aid of a perfluorinated surfactant and sonication [166]. In addition, non-linear optical limiting (one order of magnitude higher than that of GO) was identified in aqueous dispersions of FGO [167]. Such properties predispose these materials for optoelectronic and photonic applications.

## 6 Conclusions and perspectives

Fluorographene displays sharply different properties than graphene because it lacks conjugated network of π-conjugated electrons, which makes this material a wide band gap insulator. Fluorographene was born rather recently, because it was isolated in 2010, but its bulk parent graphite fluoride has been known for more than one hundred years and since the seventies GrF has been used as industrial lubricant. The industrial usage of GrF makes also easily available FG, which can be prepared from GrF by exfoliation technologies. Despite its youth, FG found several important applications in coating, batteries, separation technologies, and electrochemical sensing. Recent discoveries documenting high-reactivity of FG open new doors to a wide range of graphene derivatives. Chemical transformation of FG is easy and scalable and leads to high-yield and selective covalent graphene derivatives, like cyanographene and graphene acid. Such graphene derivatives enable reactive functional groups homogeneously distributed over the graphene surface, which can be further utilized for covalent grafting of other functionalities, e.g., carbon dots, biomacromolecules, polymers etc., to graphene. Such graphene derivatives may also function as a versatile platform for countless applications, e.g., in selective electrochemical sensing, light harvesting, catalysis etc., therefore a significant attention should be paid to synthesis and testing of these materials. The chemical processability, high-thermal stability, low surface energy, and availability make FG an extraordinary material among other 2D materials, because its properties can be turned into numerous marketed technologies in the near future.
